# Optimal timing of the cumulative dose and factors related to acute urinary toxicity in prostate cancer treated using magnetic resonance linear accelerator

**DOI:** 10.1007/s12194-026-01056-8

**Published:** 2026-04-27

**Authors:** Shohei Tanaka, Noriyoshi Takahashi, Noriyuki Kadoya, Wingyi Lee, Taichi Hoshino, Yoshiyuki Katsuta, Kazuhiro Arai, Yushan Xiao, Hisamichi Takagi, Yu Suzuki, Shinsaku Okuda, Keiichi Jingu

**Affiliations:** 1 Department of Radiation Oncology, Tohoku University Graduate School of Medicine, 1-1 Seiryo-machi, Aoba-ku, Sendai, 980-8574 Japan; 2 Department of Radiological Technology, School of Health Sciences, Faculty of Medicine, Tohoku University, Sendai, Japan

**Keywords:** Radiotherapy, MR-linac, Prostate, Unity, IPSS, Urethra toxicity

## Abstract

We investigated which timing of cumulative dose measurement is most strongly associated with acute urinary toxicity in magnetic resonance (MR)-guided linear accelerator (Linac) treatment and also explored additional contributing factors. A total of 114 patients with prostate cancer treated with five-fraction MR-Linac therapy were included. Cumulative bladder doses were calculated using deformable image registration at three time points: before treatment (pre-MRI), during position verification (PV-MRI), and after irradiation (post-MRI). Acute urinary toxicity was defined as an increase of ≥ 10 points in the International Prostate Symptom Score within 3 months from baseline. The relationships between cumulative bladder dose and acute urinary toxicity were analyzed, along with non-dosimetric contributing factors. Cumulative bladder doses calculated from PV- and post-MRI showed stronger associations with acute urinary toxicity than those from pre-MRI. The area under the curve values for bladder V37Gy (cc) and V37Gy (%) on post-MRI were 0.69 and 0.70. A higher frequency of motion correction applied for target displacement during PV-MRI was associated with a lower incidence of acute urinary toxicity (*p* = 0.04). Secondary adapt-to-shape-based recontouring and replanning shortened the MRI-to-irradiation interval, stabilized prostate positioning during irradiation, and enabled adaptation to an enlarged bladder, which may have reduced bladder dose. Therefore, cumulative bladder doses assessed on PV- and post-MRI are moderately associated with acute urinary toxicity. PV-MRI-based target motion correction and replanning may mitigate this toxicity; however, the correlation between motion correction and post-MRI bladder V37Gy (%) was weak despite statistical significance and should be interpreted with caution.

**Supplementary Information:**

The online version contains supplementary material available at 10.1007/s12194-026-01056-8.

## Introduction

Magnetic resonance–guided radiotherapy (MRgRT) is widely used for the treatment of various tumor sites [1–5]. In particular, the Elekta Unity MR-Linac (Elekta AB, Stockholm, Sweden) is a radiotherapy system that integrates a magnetic resonance imaging (MRI) unit (Philips Healthcare, Amsterdam, the Netherlands) with a 7-MV flattening filter–free (FFF) linear accelerator. This MR-guided Linac system has been implemented for prostate cancer treatment across multiple institutions [5–10].

The MR-Linac enables real-time visualization of soft tissues during treatment. Consequently, based on the anatomical information obtained from the MRI acquired on the treatment day (daily MRI), two primary adaptive strategies can be employed. The first is the adapt-to-position (ATP) approach in which only the isocenter of the treatment plan is aligned to the daily MRI without recontouring. The plan is then recalculated or re-optimized using the contours from the pre-treatment image. In other words, the adapted plan relies on the original contours, and no manual contour editing is required [11]. The second strategy, adapt-to-shape (ATS), involves recontouring based on the anatomical information from the daily MRI, followed by plan optimization accordingly [11]. In this approach, the contours are propagated from the pre-treatment image to the daily MRI using either rigid or deformable image registration (DIR), with manual adjustments applied as necessary. The treatment plan is subsequently re-optimized based on the updated contours. ATS enables treatment adaptation to inter-fractional anatomical changes observed on the daily MRI, such as rectal gas or stool displacement [12].

Despite the advantages of MR-Linac in prostate cancer radiotherapy, urinary toxicity is still reported as a treatment-related adverse effect in several studies [11–13]. The International Prostate Symptom Score (IPSS) is commonly used to assess acute urinary symptoms and evaluate treatment-related toxicity. Early identification of patients at risk for an IPSS increase following radiotherapy allows timely medical intervention. In such patients, more frequent follow-up visits and urinalysis can facilitate early detection and management of hematuria and other late-onset urinary complications.

In studies investigating prostate cancer treatment using MR-Linac, the cumulative bladder dose has been shown to correlate strongly with increases in IPSS [13, 14]. At our institution, MRI is performed three times on each treatment day—before treatment, immediately before irradiation, and after irradiation. Among these, images obtained immediately before and after irradiation most accurately represent the patient’s anatomy during irradiation. Previous studies analyzed the relationship between acute urinary toxicity and cumulative dose distributions derived from a single imaging time point within the treatment workflow (e.g., before treatment or immediately before irradiation) [13, 14]. Consequently, it remains unclear which image-based cumulative dose distribution—pre-treatment, pre-irradiation, or post-irradiation—best reflects the development of acute urinary toxicity. Establishing appropriate timing for cumulative dose calculation could help identify patients at higher risk of urinary toxicity in clinical practice, allowing risk estimation based on cumulative doses obtained at the most relevant time point.

Furthermore, the European Society for Radiotherapy and Oncology Advisory Committee on Radiation Oncology Practice consensus guidelines recommend maintaining bladder filling to protect the bowel or part of the bladder from excessive irradiation [15]. However, unlike conventional computed tomography–based treatment, MRgRT is more time-consuming. This extended workflow introduces unique challenges, including anatomical changes resulting from progressive bladder filling and positional variations due to muscle relaxation during the procedure. Only a few studies have evaluated the extent to which these factors influence bladder dose and the incidence of acute urinary toxicity.

This study had two primary objectives:

1. To identify which cumulative dose distributions—obtained before treatment, immediately before irradiation, or after irradiation—and corresponding dose constraints are most strongly associated with acute urinary toxicity.
2. To investigate the relationship between factors contributing to anatomical changes specific to MRgRT and their impact on bladder dose and acute urinary toxicity.

## Materials and methods

### Patient characteristics

This study included 189 patients with prostate cancer who underwent MR-Linac treatment. Of these, 17 patients without post-irradiation MRI, 15 whose treatment was interrupted due to MR-Linac malfunction, 21 with baseline IPSS > 20, 8 who received only two fractions, 1 who received a prescribed dose of 35 Gy, and 13 with large bladder-volume variations per fraction resulting in significant errors in DIR for dose accumulation were excluded. Consequently, 114 patients were included in the final analysis (low- or intermediate-risk group, *n* = 67; high-risk group, *n* = 47). Detailed patient characteristics are provided in Supplementary Table S1. None of the high-risk patients received pelvic lymph node irradiation. Low- or intermediate-risk patients were treated with 36.25 Gy in five fractions. High-risk patients received 36.25 Gy to the planning target volume (PTV) and 40 Gy to the clinical target volume (CTV) in five fractions. Nearly all patients underwent SpaceOAR (Boston Scientific Corporation, Marlborough, MA, USA) hydrogel insertion to create a separation between the prostate and rectum. IPSS assessments were performed on the first treatment day, once during treatment, and once or twice at 1–3-month intervals following treatment initiation. Data were collected using patient self-administered questionnaires.

This study was approved by the institutional ethics committee. Given its retrospective design, the requirement for informed consent was waived. All procedures were conducted in accordance with institutional and national ethical standards and regulations.

### Organs-at-risk (OARs) and target contouring on MRI simulation

Reference plans were created on the simulation MRI for all patients. In the low-risk group, the CTV encompassed the prostate. In the intermediate- and high-risk groups, the CTV included both the prostate and the seminal vesicles, extending 1.5 cm from the prostate base into the seminal vesicles. The PTV margin was generally set at 5 mm in all directions. However, for patients in whom meeting bladder dose constraints was deemed difficult by the radiation oncologist or medical physicist, the superior margin was reduced from 5 mm to 3 mm. Radiation oncologists delineated the rectum, femoral heads, urethra, small bowel, and bladder as organs-at-risk (OARs). The entire bladder (including the lumen) was contoured, rather than the bladder wall alone. The urethra was contoured with a nominal diameter of 2 mm, and a 2-mm planning OAR volume (PRV) margin was added, resulting in a total diameter of 6 mm.

### Treatment planning for simulation MRI

Treatment planning was performed using the Monaco treatment planning system (Elekta AB, Stockholm, Sweden). Electron density values were assigned to the CTV, bladder, rectum, bilateral femoral heads, bone, and body contour. A step-and-shoot intensity-modulated radiotherapy plan employing nine beams was generated. Dose calculations were performed using the Graphics Processing Unit–based Monte Carlo algorithm, with a calculation grid size of 3 mm for the low- and intermediate-risk groups and 2.5 mm for the high-risk group, and with a statistical uncertainty of 1%. A calculation grid size of 2.5 mm was used for high-risk patients to facilitate compliance with the maximum urethral PRV dose constraint of < 40 Gy. However, excessively small grid sizes substantially increased optimization time; therefore, a 2.5 mm grid size was used to balance good dose distribution with computational efficiency. A 7-MV FFF X-ray beam was used for all plans. The maximum number of segments per plan was limited to 60 for low- and intermediate-risk patients and 100 for high-risk patients. The minimum monitor units per segment, minimum segment width, fluence smoothing, plan quality, and minimum segment area were set to 5, 0.5 cm, low, 5, and 4 cm^2^, respectively. Institutional dose constraints are summarized in Supplementary Tables S2 (low- or intermediate-risk group) and S3 (high-risk group).

### Online adaptive radiotherapy (ART) workflow

**Fig. 1:**
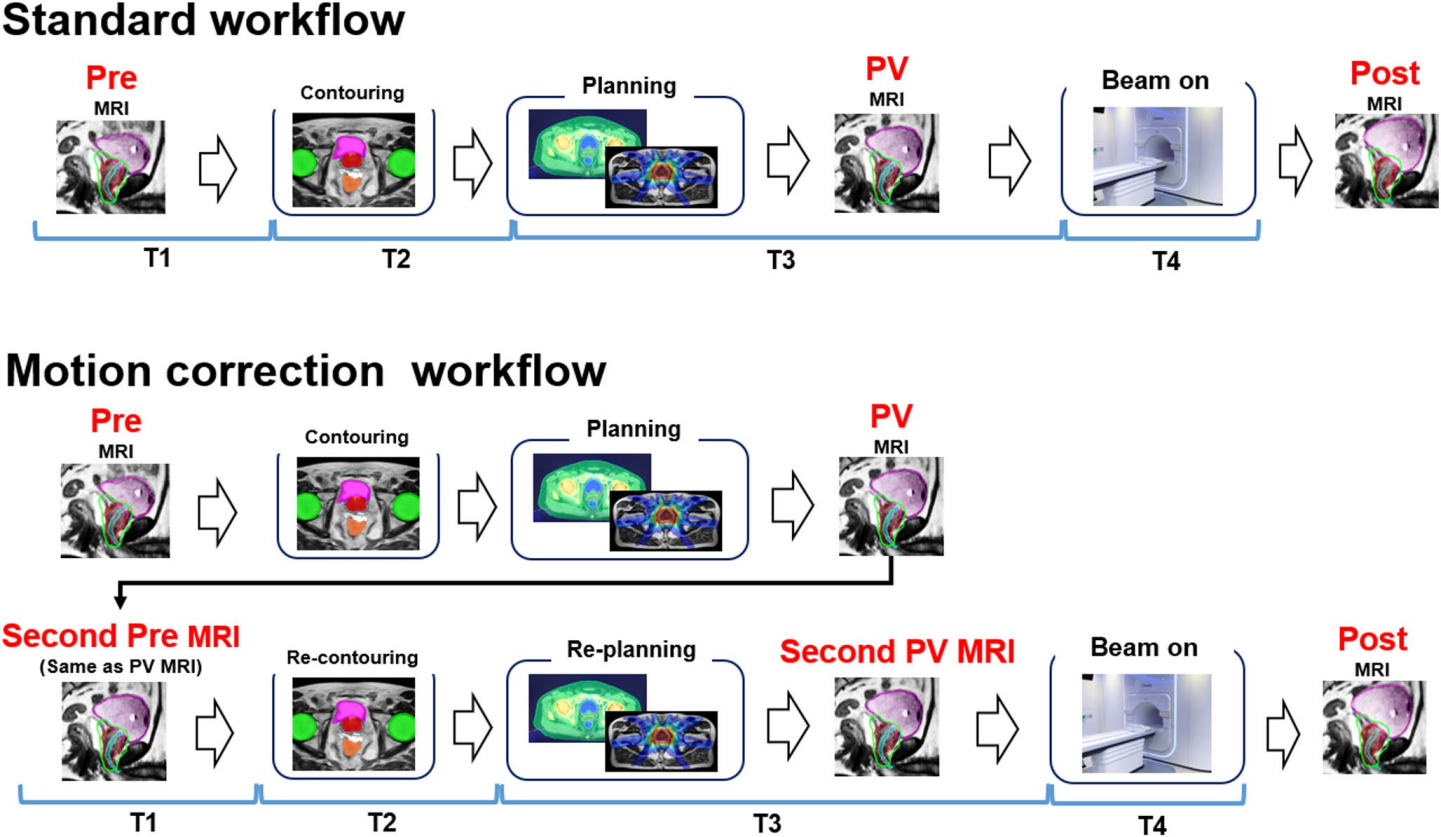
Workflow of adaptive radiotherapy for patients with prostate cancer treated at our institution. Standard workflow: Pre-magnetic resonance imaging (pre-MRI) refers to the first MRI performed on treatment day, which is used to create the treatment plan. After contouring and planning, position verification (PV)-MRI is acquired immediately before irradiation to confirm whether the target is positioned correctly within the planning target volume. Post-MRI is obtained immediately after irradiation to assess for any movement of the target or organs at risk during irradiation. Motion correction workflow: If target displacement is detected on the first PV-MRI, the PV-MRI is treated as a second pre-MRI, and recontouring and replanning are performed on the second pre-MRI. Subsequently, the target position is reevaluated using a second PV-MRI, and irradiation is delivered if no displacement is observed. Post-MRI is acquired after irradiation. T1–T4 represent time intervals for each process: T1 indicates the time from pre-MRI acquisition to contouring, T2 from contouring to planning, T3 from planning to irradiation, and T4 the irradiation time

The standard bladder-filling protocol consisted of drinking 200 mL of water approximately 30 min before treatment initiation. The online adaptive radiotherapy (ART) workflow used at our institution is illustrated in Fig. 1. In this study, most patients underwent an initial ATS workflow on the treatment planning MRI before position verification, while one patient underwent an initial ATP workflow. Figure 1 illustrates the ATS workflow, representing our institution’s primary ART workflow. At the start of each treatment session, an MRI scan was performed and defined as the pre-MRI. The structures delineated on the simulation MRI were transferred to the pre-MRI via DIR, after which radiation oncologists manually reviewed and corrected the target and OAR contours to generate clinically acceptable structures. A treatment plan was then created based on the pre-MRI. Because prostate motion can occur during treatment preparation, an additional MRI was acquired immediately before irradiation, referred to as the position verification (PV) MRI. If target displacement was observed on the PV-MRI, plan adaptation was performed using an additional ATS procedure to correct for the motion. In this study, this sequence of pre-irradiation position adjustments was collectively defined as motion correction, which was performed using ATS in most patients. Motion correction encompasses all the involved procedures, including re-fusion of targets with positional displacement on PV-MRI, recontouring of the target and OARs, and treatment replanning using ATS optimization. No predefined quantitative threshold was established for motion correction of prostate displacement; implementation was determined by the physician. Specifically, motion correction was performed when the target was near the PTV boundary on PV-MRI and was likely to move outside the PTV during irradiation. When motion correction was required, the first PV-MRI was redefined as “the second pre-MRI,” and a subsequent MRI acquired for repeated PV was designated as “the second PV-MRI.” The ATS adaptive workflow loop triggered by motion correction required recontouring and re-optimization of the treatment plan on the second pre-MRI (hereafter referred to as secondary ATS-based recontouring and re-optimization), thereby extending the overall time spent on the treatment couch. If no prostate motion was detected on the PV-MRI, irradiation proceeded using the existing plan. The MRI acquired immediately after irradiation was defined as the post-MRI.

### Contouring and dose recalculation for PV- and post-MRI

Structural data and dose distributions were not inherently available for the PV- and post-MRI scans. Therefore, the structures delineated on the pre-MRI were transferred to the PV- and post-MRI using DIR. Medical physicists subsequently reviewed and modified the OAR and target contours as necessary to complete the structure sets on the PV- and post-MRI. The treatment plan information was also transferred to both the PV- and post-MRI datasets. Dose distributions were then reproduced on these images by recalculating the treatment dose using the same radiation fields and isocenter positions as those in the original treatment plan, without any modification. This dose recalculation was performed using the “original segments” function in the Monaco treatment planning system.

### Creation of cumulative dose distribution

Cumulative dose calculations were performed using the RayStation software, Version 6.2.0 (RaySearch Laboratories, Stockholm, Sweden). Hybrid DIR employing the ANACONDA algorithm was used for dose accumulation [16]. This algorithm integrates both intensity-based and structure-based DIR methods. The CTV, bladder, urethra PRV, and rectum were defined as control regions of interest (ROIs), and DIR registration was centered on these ROIs. The DIR calculation grid was set to 2.5 mm. Each MRI from fractions 2–5 was registered to the MRI of fraction 1. The dose distributions from fractions 2–5 were then deformed according to the corresponding vector fields. Finally, the cumulative dose across all five fractions was generated on the MRI from fraction 1. This process was repeated for each of the three MRI types: pre-, PV-, and post-MRI. To verify the accuracy of the deformation, the average Dice similarity coefficient (DSC) between the deformed bladder (based on vector fields from MRI fractions 2–5) and the true bladder in MRI fraction 1 was calculated. Additionally, the average 95% Hausdorff distance was calculated as a supplementary measure of registration accuracy.

### Extraction of dose constraints and evaluation

From the cumulative bladder dose distributions, the percentage volume and absolute volume (cc) receiving doses ≥ 10 Gy (V10Gy), V15Gy, V18.1 Gy, V25Gy, V30Gy, V35Gy, V37Gy, and V40Gy, as well as the maximum dose (Dmax), were extracted. V18.1 Gy and V37Gy were specifically included in the analysis because they represent institutional dose constraints (Supplementary Tables S2 and S3). In accordance with previous studies, patients exhibiting an increase in IPSS of ≥ 10 points from baseline within 3 months after radiotherapy were defined as having clinically significant acute urinary toxicity [13, 14]. The ability of each dose constraint to predict acute urinary toxicity was assessed using receiver operating characteristic analysis, and predictive performance was quantified by the area under the curve (AUC).

### Extraction of factors other than dose constraints and evaluation

In addition to bladder dose constraints, specific factors related to the online ART workflow were extracted and evaluated for their association with acute urinary toxicity. The factors analyzed included prostate movement, bladder volume, bladder filling, irradiation time, online ART workflow time, and the number of motion corrections. Online ART workflow time was defined as the interval from pre-MRI acquisition start to irradiation start (T1–T3 in Fig. 1). In the ATS workflow loop triggered by motion correction, the online ART workflow time spanned from the start of acquisition of the second pre-MRI (first PV-MRI) to the start of irradiation (T1–T3 of motion correction workflow in Fig. 1). The number of motion corrections was defined as the total number of times motion correction was applied across the five ART workflows (range, 0–5). Detailed descriptions and calculation methods for these parameters are provided in Supplementary Table S4.

Additionally, clinical factors previously reported to be associated with urinary toxicity were extracted, including age [17], baseline IPSS [18–20], T stage [19], prostate volume [17], smoking status [17], and diabetes [17]. Herein, we also included prostate-specific antigen level, neoadjuvant chemotherapy, and risk group classification and evaluated their associations with acute urinary toxicity.

Univariate analyses were conducted to assess associations between potential factors and acute urinary toxicity. The Mann–Whitney *U* test was used for continuous variables, and the chi-square test was applied for categorical variables. Spearman’s correlation coefficients were calculated to evaluate multicollinearity among the variables. Factors with *p* < 0.1 in the univariate analysis were further examined for correlations with bladder dose indices to identify potential confounding factors. Only those with *p* < 0.1 and not exhibiting significant collinearity were included in the multivariate analysis, which was performed using linear regression.

### Additional analyses regarding motion correction

Additional analyses were conducted to evaluate the impact of the number of motion corrections on bladder dose. Initially, correlations between the number of motion corrections and bladder dose constraints for the PV- and post-MRI datasets were examined using Spearman’s correlation coefficients. Furthermore, to explore the underlying mechanisms of these associations, a series of hypothesis-driven analyses were performed.

Hypothetically, secondary ATS-based recontouring and re-optimization with motion correction (repeating the ART process) offers three potential advantages for reducing bladder dose.

First, longer treatment couch time relaxes patients, which may lead to reduced body movement during irradiation. Repeating the ART workflow involves recontouring the displaced target and re-optimizing the treatment plan. Although this extends the time the patient is on the couch, patient motion decreases as the session progresses, resulting in more stable positioning before irradiation. Reduced motion limits bladder displacement into high-dose regions and may consequently decrease bladder dose exposure. To test this hypothesis, inferior and posterior prostate displacements during irradiation were compared between treatment fractions with and without motion correction. Prostate motion during irradiation was defined as the displacement of the prostate between the PV-MRI and post-MRI. To further assess the relationship between prostate motion and bladder dose escalation during irradiation, Spearman’s correlation analyses were performed between posterior and inferior prostate movements and the increase in bladder dose across all treatment fractions. The increase in bladder dose during irradiation was defined as the difference between bladder doses measured on the PV-MRI and post-MRI. The sample size included all treatment fractions, resulting in a total of 570 fractions (114 patients × 5 fractions).

Second, secondary ATS-based recontouring on the second pre-MRI may require less time than the initial contouring, potentially shortening the interval between MRI acquisition and irradiation (T1–T3 in Fig. 1). In the ATS workflow, initial ATS-based contouring without motion correction uses structure data propagated via DIR from the simulation contours to the daily MRI. Because these MRIs are acquired on different days, anatomical changes often require substantial modifications. For secondary ATS-based recontouring with motion correction, contours from the immediately preceding pre-MRI can be propagated to the second pre-MRI using DIR. As the anatomy is largely unchanged, only minor adjustments are typically needed. This reduces recontouring time and shortens the interval from the second pre-MRI to irradiation compared to sessions without motion correction. A shorter MRI-to-irradiation interval may help mitigate anatomical changes in the bladder occurring over time, thereby reducing bladder dose exposure. To test this hypothesis, the MRI-to-irradiation interval was compared between treatment fractions with and without motion correction. To ensure a fair comparison of the MRI-to-irradiation interval, only treatment fractions utilizing the ATS workflow were analyzed. The interval was defined as the duration from the second pre-MRI to irradiation for fractions with motion correction and from pre-MRI to irradiation for those without motion correction (T1–T3 in Fig. 1). Additionally, correlations between the MRI-to-irradiation interval and the increase in bladder dose from pre- to PV-MRI were analyzed using Spearman’s correlation coefficients. This analysis included all treatment fractions from 114 patients (570 fractions: 114 patients × 5 fractions). Fractions with missing time measurement data or those using ATP for the ART workflow were excluded, resulting in a final analysis cohort of 499 fractions. Of these, motion correction was applied in 112 fractions, while 387 fractions were delivered without motion correction.

Third, bladder dose may be reduced through secondary ATS-based re-optimization, adjusting the dose distribution to the enlarged bladder shape. Three treatment plans were retrospectively developed to test this hypothesis. For the first plan, the initial plan generated on the pre-MRI using the ATS workflow was not modified; however, the dose was recalculated on the PV-MRI using the same radiation field segments and fixed monitor units. This approach simulated a scenario in which, despite bladder changes due to filling between the pre-MRI and PV-MRI, no secondary ATS-based re-optimization was conducted, and the initial plan created on the pre-MRI was delivered to the bladder on the PV-MRI unchanged. This plan was defined as the “plan without replanning.” The second plan was re-optimized on the second pre-MRI, adjusting the dose distribution to the enlarged bladder via a secondary ATS-based re-optimization applied after motion correction. This was deemed as “replanning with ATS.” The third plan replaced the second plan’s ATS-based optimization with ATP-based re-optimization after applying motion correction. This approach was described as “replanning with ATP.” Bladder dose constraints were compared between these three plans. In this analysis, 10 patients were retrospectively selected from a cohort of 114 patients. These 10 patients exhibited minimal posterior and inferior prostate movements (average posterior and inferior displacements of 0.8 mm and − 0.2 mm, respectively, between pre- and PV-MRI) and rapid bladder filling (average pre-PV bladder volume increase of 66 cc [96%]). Among them, five patients were in the low- or intermediate-risk group, whereas five were in the high-risk group. Statistical comparisons were performed using the Wilcoxon signed-rank test.

All statistical analyses were performed using MATLAB (MathWorks, Inc., Natick, MA, USA), and a *p* value < 0.05 was considered statistically significant.

## Results

The mean DSCs used to evaluate the accuracy of deformation between the deformed and true bladder were 0.97 ± 0.03 (range, 0.85–0.99), 0.98 ± 0.02 (range, 0.88–0.99), and 0.98 ± 0.02 (range, 0.84–0.99) for the pre-, PV-, and post-MRI, respectively. The average DSC values for all patients met the recommended tolerance of > 0.8, as specified in the American Association of Physicists in Medicine Task Group No. 132 [21]. The mean 95% Hausdorff distances were 3.0 ± 2.0 (range, 1.6–13.5), 2.7 ± 1.6 (range, 1.5–12.4), and 2.9 ± 2.1 (range, 1.6–13.2) mm for the pre-, PV-, and post-MRI, respectively. In nine patients, at least one fraction exhibited misregistration (DSC < 0.8) due to substantial differences in bladder volume between MRI fraction 1 and fractions 2–5. Inspection of these cases revealed cranially located discrepancies. A mean difference of 4.1 ± 1.2 cm was observed between the cranial edge of the bladder and of the deformed bladder. The mean bladder volumes on MRI fraction 1 and deformed bladder volumes were 123.8 ± 38.5 cm³ and 231.1 ± 92.8 cm³, respectively. This volumetric difference was observed only in the cranial direction. Therefore, consistent with previous studies, these patients were retained in the analysis, as the effects of misregistration were confined to regions outside the medium- to high-dose regions [14].

**Fig. 2:**
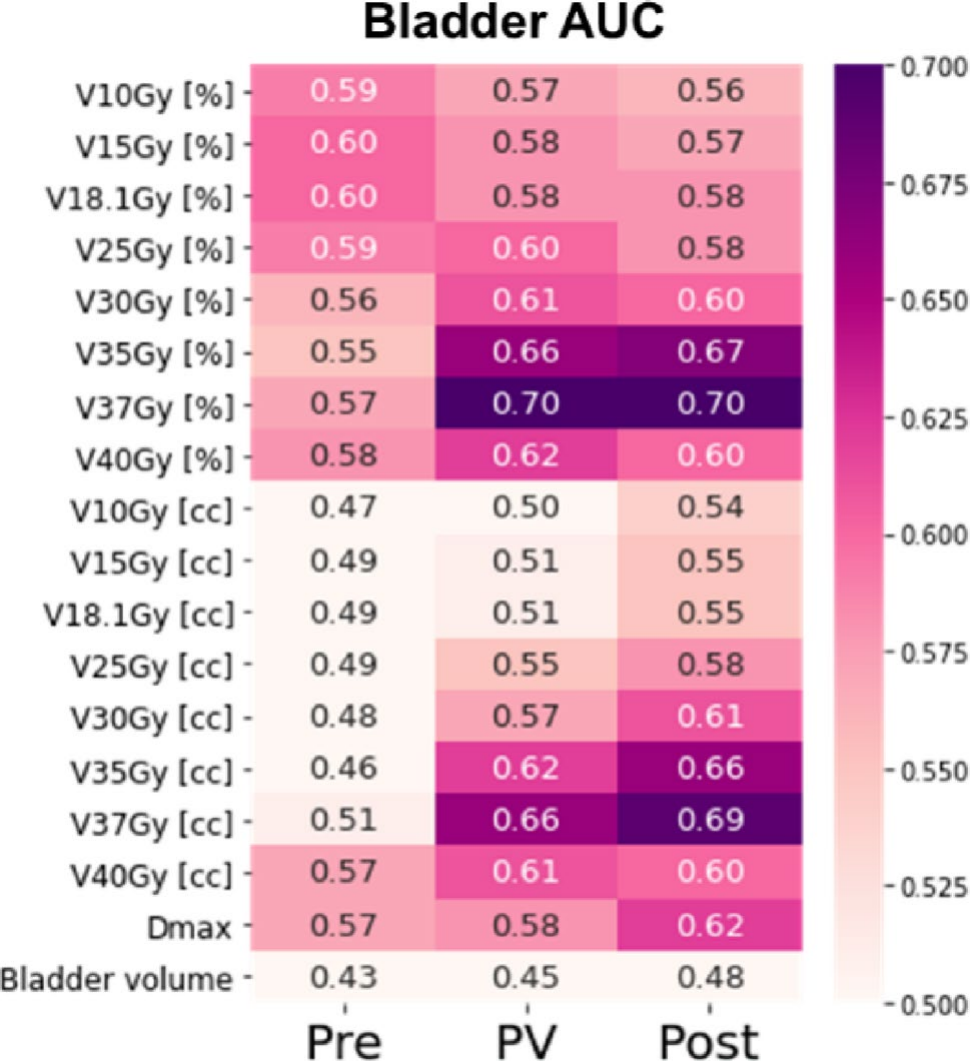
Area under the curve (AUC) heatmap depicting the predictive performance of each dose constraint for acute urinary toxicity, based on cumulative bladder doses from pre-, position verification (PV)-, and post-magnetic resonance imaging (MRI). The AUC values for bladder volume are also shown. Higher and lower AUCs are represented by dark and light magenta, respectively. The percentage and absolute bladder volumes receiving doses ≥ 37 Gy (V37Gy) in the PV- and post-MRI datasets demonstrated the highest predictive performance

Figure 2 presents the AUC values for the bladder dose constraints. The AUCs were higher for the PV- and post-MRI than for the pre-MRI. Among the PV- and post-MRI datasets, higher-dose parameters (V35Gy–V40Gy) demonstrated greater AUCs than lower dose parameters (V10Gy–V30Gy). In particular, bladder V37Gy (%) and V37Gy (cc) achieved relatively high AUCs in the post-MRI (0.70 and 0.69, respectively) and PV-MRI (0.70 and 0.66, respectively). Bladder volume on the pre-, PV-, and post-MRI showed no significant association with acute urinary toxicity (AUC = 0.43, 0.45, and 0.48, respectively).

Table 1 summarizes the results of the univariate analysis assessing factors other than dose constraints in relation to acute urinary toxicity. Factors with p-values less than 0.1 in the univariate analysis are shown in bold. Variables with *p* < 0.1 in the univariate analysis included the number of motion corrections (*p* = 0.04), inferior prostate movement (PV–post) (*p* = 0.08), bladder filling (%) from pre- to PV-MRI (*p* = 0.05), age (*p* = 0.07), T stage (*p* = 0.08). Correlations between these variables and post-MRI bladder V37Gy are presented in Supplementary Table S5. Inferior prostate movement (PV–post), age, and T stage were significantly correlated with post-MRI bladder V37Gy (%) (*p <* 0.001, = 0.03, and < 0.001, respectively), indicating potential confounding effects; hence, they were excluded from the multivariate analysis. Although the number of motion corrections and bladder filling (%) (pre-PV) were also correlated with post-MRI bladder V37Gy (%) (*p* = 0.02, 0.04, respectively), these were retained for multivariate analysis because they were considered predictors influencing bladder V37Gy, which served as a mediating factor. Supplementary Figure S1 illustrates the correlations among all variables included in the univariate analysis. No significant collinearity was observed between the number of motion corrections and bladder filling (%) (pre-PV) (*r* = − 0.04, *p* = 0.67). In the multivariate analysis including these two factors, only the number of motion corrections remained significant (*p* = 0.04).

**Table 1:** Univariate and multivariate analyses of factors other than dose constraints associated with acute urinary toxicity

Factors	Odds ratio	Univariate analysis		Multivariate analysis
		Mann–Whitney U test *p* value	Chi-square test *p* value	*p* value
Number of motion corrections	0.76	**0.041**		**0.035**
Irradiation time [min]	1.065	0.725		
Online ART workflow time [min]	1.04	0.138		
Posterior prostate movement [mm] (Pre→PV)	1.03	0.820		
Posterior prostate movement [mm] (PV→Post)	1.474	0.285		
Posterior prostate movement [mm] (Pre→Post)	1.235	0.516		
Inferior prostate movement [mm] (Pre→PV)	0.92	0.912		
Inferior prostate movement [mm] (PV→Post)	1.235	**0.079**		
Inferior prostate movement [mm] (Pre→Post)	1.263	0.224		
Bladder volume [cc] (Pre)	0.995	0.242		
Bladder volume [cc] (PV)	0.998	0.420		
Bladder volume [cc] (Post)	0.999	0.735		
Bladder filling [cc] (Pre→PV)	1.003	0.338		
Bladder filling [cc] (PV→Post)	1.003	0.539		
Bladder filling [cc] (Pre→Post)	1.002	0.413		
Bladder filling [%] (Pre→PV)	3.102	**0.050**		0.157
Bladder filling [%] (PV→Post)	2.809	0.315		
Bladder filling [%] (Pre→Post)	1.545	0.105		
Age [years]	1.063	**0.074**		
IPSS baseline	1.03	0.222		
Prostate volume [cc]	1.015	0.920		
PSA level	0.996	0.354		
Diabetes (yes vs. no)	0.626		0.451	
Smoking (yes vs. no)	0.808		0.740	
Neoadjuvant chemotherapy (yes vs. no)	1.271		0.555	
T stage	1.095		**0.084**	
Risk group (low vs. intermediate vs. high)	1.127		0.118	

ART: adaptive radiation therapy; PV: position verification; IPSS: International Prostate Symptom Score; PSA: prostate-specific antigen

Supplementary Figure S2 shows a histogram of motion correction application frequencies among the 114 patients. The most common category was zero applications, indicating that motion correction was not applied, which was observed in 49 patients. In contrast, motion correction was applied in all fractions (five applications) in five patients. The mean number of motion correction applications per patient was 1.35 (median: 1). Table 2 summarizes the correlations between the number of motion corrections and bladder dose constraints for the PV- and post-MRI datasets. p-values indicating a statistically significant difference are shown in bold. Weak negative correlations were observed between the number of motion corrections and post-MRI bladder V37Gy (%) and V37Gy (cc) (*r* = − 0.23 and − 0.19, respectively), both of which were statistically significant (*p* = 0.015 and 0.039, respectively). Furthermore, all post-MRI bladder dose constraints exhibited a negative trend with the number of motion corrections, suggesting that a higher frequency of motion corrections tended to decrease post-MRI bladder doses. The following sections present the results of analyses designed to verify the three hypotheses proposed to explain this relationship.

**Table 2:** Correlation between bladder dose constraints on PV- and post-MRI and the number of motion corrections

Structure	Timing	Variable	Spearman’s correlation coefficient	*p* value
Bladder	PV-MRI	V10Gy (%)	−0.11	0.227
		V15Gy (%)	−0.09	0.335
		V18.1 Gy (%)	−0.05	0.582
		V25Gy (%)	0.00	0.988
		V30Gy (%)	0.02	0.830
		V35Gy (%)	0.00	0.981
		V37Gy (%)	−0.06	0.533
		V40Gy (%)	−0.06	0.530
		V10Gy (cc)	−0.06	0.561
		V15Gy (cc)	−0.05	0.609
		V18.1 Gy (cc)	−0.03	0.773
		V25Gy (cc)	−0.01	0.934
		V30Gy (cc)	0.02	0.870
		V35Gy (cc)	0.03	0.783
		V37Gy (cc)	−0.01	0.954
		V40Gy (cc)	−0.05	0.572
		Dmax	0.00	0.999
	Post-MRI	V10Gy (%)	−0.14	0.142
		V15Gy (%)	−0.12	0.221
		V18.1 Gy (%)	−0.10	0.277
		V25Gy (%)	−0.10	0.295
		V30Gy (%)	−0.13	0.166
		V35Gy (%)	−0.22	**0.021**
		V37Gy (%)	−0.23	**0.015**
		V40Gy (%)	−0.13	0.174
		V10Gy (cc)	−0.08	0.371
		V15Gy (cc)	−0.08	0.416
		V18.1 Gy (cc)	−0.07	0.464
		V25Gy (cc)	−0.07	0.433
		V30Gy (cc)	−0.10	0.302
		V35Gy (cc)	−0.14	0.127
		V37Gy (cc)	−0.19	**0.039**
		V40Gy (cc)	−0.11	0.232
		Dmax	−0.11	0.264

MRI: magnetic resonance imaging; PV: position verification; VXXGy (cc): absolute bladder volume (cc) receiving ≥ XX Gy, VXXGy (%): percentage of bladder volume receiving ≥ XX Gy, Dmax: maximum bladder dose

**Fig. 3:**
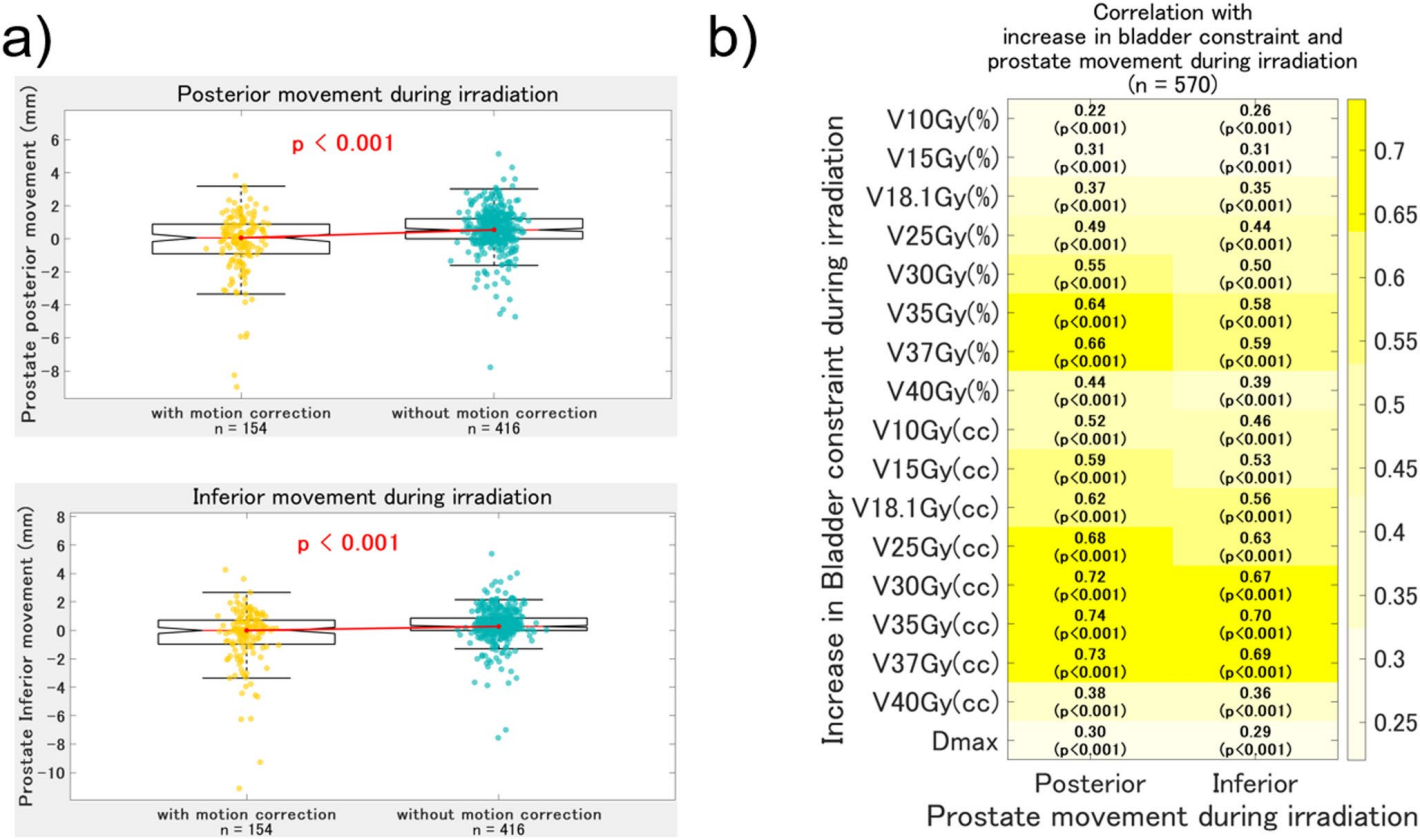
Relationship between motion correction and posterior and inferior prostate movements during irradiation (a),and heatmap of Spearman’s correlation coefficients showing the relationship between the increase in bladder dose during irradiation and posterior and inferior prostate movements (b) In Fig. 3a, all treatment fractions are plotted. Both posterior and inferior prostate movements were significantly smaller with motion correction than without motion correction (*p* < 0.001 for both). In Fig. 3b, weak to moderate correlations were observed across all bladder dose constraints, indicating that greater posterior and inferior prostate displacements tended to increase bladder dose. Moderate correlations were specifically observed between the increase in bladder volume receiving ≥ 37 Gy (V37Gy [cc]) and posterior (*r* = 0.73) and inferior (*r* = 0.69) prostate movements

The first hypothesis was that although repeating the ART workflow, including recontouring and re-optimization associated with motion correction, prolongs time on the treatment couch, prostate motion becomes more stable, reducing displacement during irradiation. Figure 3a compares posterior and inferior prostate displacements during irradiation between fractions with and without motion correction. Both posterior and inferior prostate movements were significantly smaller in the motion correction group than in the non-motion correction group (Mann–Whitney *U* test, *p* < 0.001 for both). The relationship between prostate movement and the increase in bladder dose during irradiation is illustrated in Fig. 3b. Moderate positive correlations were observed between the increase in bladder V37Gy (cc) and posterior (*r* = 0.73) and inferior (*r* = 0.69) prostate displacements, indicating that suppression of prostate motion during irradiation contributes to a reduction in bladder dose.

**Fig. 4:**
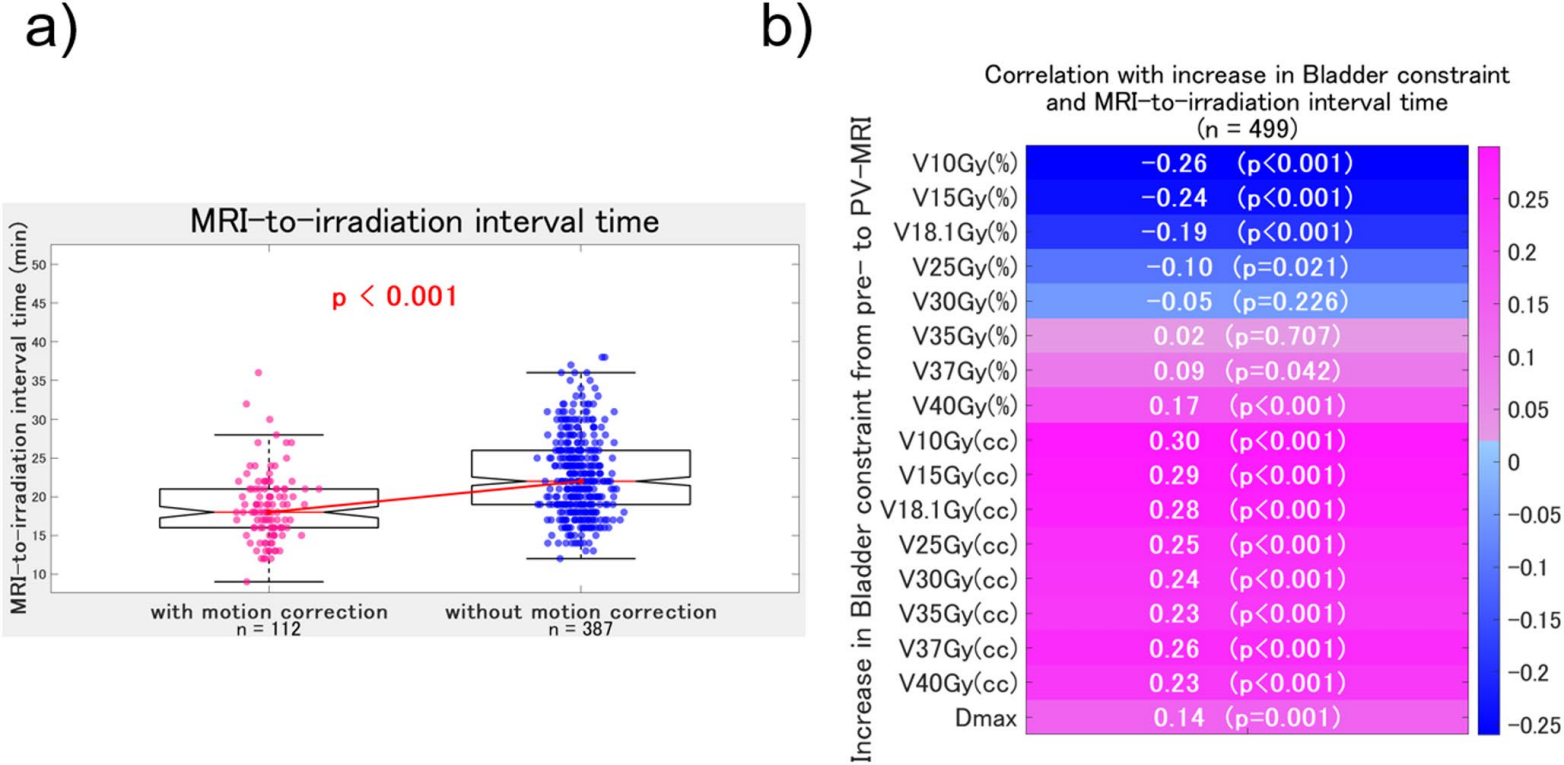
Comparison of the interval between magnetic resonance imaging (MRI) acquisition and irradiation with and without motion correction (a), and correlation between the MRI-to-irradiation interval and the increase in bladder dose from pre- to position verification (PV) MRI (b). In Fig. 4a, the MRI-to-irradiation interval was defined as the time from the second pre-MRI (used for plan re-creation) to irradiation in the motion correction group and from the pre-MRI to irradiation in the group without motion correction. Fractions with motion correction showed a significantly shorter MRI-to-irradiation interval than those without motion correction (*p* < 0.001). In Fig. 4b, a weak positive correlation was observed between the MRI-to-irradiation interval and the increase in bladder volume receiving ≥ 37 Gy (V37Gy [cc]) (*r* = 0.26, *p* < 0.001), indicating that a shorter interval may contribute to a reduction in bladder V37Gy (cc)

The second hypothesis was that secondary ATS-based recontouring requires less time than initial pre-MRI contouring, resulting in a shorter interval between MRI acquisition and irradiation. Supplementary Figure S3 shows a breakdown of each time component within the online ART workflow, including fractions wherein the ATS workflow was applied. In fractions where motion correction triggered the ATS adaptive workflow loop, a substantial decrease in contouring time led to a shorter MRI-to-irradiation interval (T2 in Supplementary Figure S3). Figure 4a compares interval between MRI acquisition and the start of irradiation for fractions with and without motion correction. Treatment fractions with motion correction showed a significantly shorter MRI-to-irradiation interval than those without motion correction (*p* < 0.001). The relationship between the MRI-to-irradiation interval and the increase in bladder dose from pre- to PV-MRI is shown in Fig. 4b. A weak positive correlation was observed for bladder V37Gy (cc) (*r* = 0.26, *p* < 0.001), indicating that a shorter MRI-to-irradiation interval may contribute to a reduction in bladder V37Gy (cc).

The third hypothesis proposed that secondary ATS-based re-optimization reduces bladder dose by adjusting dose distribution to the enlarged bladder shape. Table 3 summarizes the bladder dose constraints for three plans: plan without replanning (the initial plan on pre-MRI was delivered to the enlarged bladder of PV-MRI without modification), replanning with ATS (the plan was re-optimized using ATS to accommodate the enlarged bladder), and replanning with ATP (the plan was re-optimized using ATP). p-values indicating a statistically significant difference are shown in bold. Higher-dose parameters (V30Gy–V37Gy) were significantly lower in replanning with ATS than in no replanning and replanning with ATP (*p* < 0.05). Supplementary Figure S4 illustrates these plans for a representative patient. Initially, V30Gy (magenta line) and V37Gy (orange line) were optimized to conform to the bladder in the pre-MRI; however, as bladder volume on the PV-MRI increased, the bladder received a higher radiation dose in the plan without replanning. Re-optimization on the PV-MRI reduced the radiation dose to the bladder, as shown by the ATS replanning.

**Table 3:** Average bladder dose constraints for 10 patients. Plan without replanning involved recalculating the pre-MRI plan on PV-MRI, and the plan with replanning entails re-optimizing on PV-MRI

Structure	Variable	Plan without replanning	Replanning with ATP	Replanning with ATS	*p* value (Plan without replanning vs. Replanning with ATS)	*p* value (Replanning with ATP vs. Replanning with ATS)
Bladder	V10Gy (%: mean ± SD)	54.2 ± 21.6	53.4 ± 21.5	54.8 ± 20.6	0.625	0.232
	V15Gy (%: mean ± SD)	36.2 ± 12.5	35.6 ± 12.2	36.0 ± 12.3	1.000	0.770
	V18.1 Gy (%: mean ± SD)	27.9 ± 8.2	27.6 ± 8.1	26.5 ± 7.5	0.105	0.193
	V25Gy (%: mean ± SD)	16.0 ± 3.2	16.0 ± 3.2	14.4 ± 3.1	**0.027**	**0.037**
	V30Gy (%: mean ± SD)	10.6 ± 2.0	10.6 ± 1.9	9.2 ± 2.2	**0.020**	**0.014**
	V35Gy (%: mean ± SD)	5.7 ± 1.4	5.8 ± 1.3	4.6 ± 1.5	**0.010**	**0.014**
	V37Gy (%: mean ± SD)	3.1 ± 1.2	3.3 ± 1.2	2.2 ± 1.0	**0.002**	**0.002**
	V40Gy (%: mean ± SD)	0.4 ± 0.5	0.5 ± 0.6	0.2 ± 0.3	0.125	0.063
	V10Gy (cc: mean ± SD)	73.8 ± 24.6	72.7 ± 24.4	75.0 ± 23.9	0.625	0.232
	V15Gy (cc: mean ± SD)	49.4 ± 14.1	48.5 ± 13.6	49.4 ± 14.4	1.000	0.846
	V18.1 Gy (cc: mean ± SD)	38.1 ± 9.4	37.7 ± 9.1	36.4 ± 8.9	0.131	0.193
	V25Gy (cc: mean ± SD)	22.1 ± 4.3	22.0 ± 4.2	19.9 ± 4.5	0.049	0.084
	V30Gy (cc: mean ± SD)	14.6 ± 3.4	14.7 ± 3.3	12.8 ± 3.7	**0.020**	**0.020**
	V35Gy (cc: mean ± SD)	8.0 ± 2.6	8.1 ± 2.5	6.4 ± 2.5	**0.014**	**0.010**
	V37Gy (cc: mean ± SD)	4.2 ± 1.9	4.5 ± 1.7	2.9 ± 1.5	**0.002**	**0.002**
	V40Gy (cc: mean ± SD)	0.5 ± 0.7	0.6 ± 0.8	0.3 ± 0.3	0.125	0.063
	Dmax (Gy: mean ± SD)	40.5 ± 1.8	40.6 ± 1.9	39.8 ± 1.7	**0.002**	**0.002**

MRI: magnetic resonance imaging; PV: position verification; VXXGy (cc): absolute bladder volume (cc) receiving ≥ XX Gy; VXXGy (%): percentage of bladder volume receiving ≥ XX Gy; Dmax: maximum bladder dose; SD: standard deviation

## Discussion

This study investigated which time points of cumulative dose distribution were most closely associated with acute urinary toxicity. Additionally, other contributing factors were analyzed. The results indicated that the cumulative dose distributions derived from PV- and post-MRI were more strongly associated with increases in IPSS than those from pre-MRI (Fig. 2). This is because they capture the anatomy immediately before and after irradiation, reflecting the actual treatment geometry. There was no substantial difference in predictive accuracy between cumulative doses calculated from PV- and post-MRI, and both showed good accuracy (Fig. 2). Therefore, calculating cumulative doses based on PV- or post-MRI may identify patients at an early stage of increased risk for urinary toxicity. However, even the highest predictive performance observed in the present study yielded an AUC of 0.70, which is insufficient for robust individual risk prediction in clinical practice. Therefore, future studies should improve predictive accuracy by incorporating additional predictive factors. The integration of cumulative dose information derived from PV- or post-MRI as supplementary data, alongside imaging-based features such as radiomics and clinical factors including blood biomarkers, may enhance predictive performance.

PV-MRI bladder V37Gy (%) and post-MRI V37Gy (%) achieved the highest predictive performance, with an AUC of 0.7 (Fig. 2). Bohoudi et al. reported that bladder V20Gy(cc)–V32.6 Gy(cc) across five fractions were associated with acute urinary toxicity [13]. Similarly, Willigenburg et al. found that bladder V10Gy–V35Gy(%) correlated with acute urinary toxicity [14]. Although their findings differ slightly from ours, showing associations at lower and intermediate dose levels, the discrepancy may be attributable to differences in patient cohorts. Specifically, their analyses primarily included low- and intermediate-risk patients, whereas the present study also involved high-risk patients who received higher radiation doses. Consistent with this, prior studies that included high-risk patients demonstrated that higher doses, such as V90Gy and V75Gy, to the bladder trigone were significantly associated with urinary toxicity [22, 23]. In the current study, V37Gy showed the strongest association with urinary toxicity, corresponding to equivalent doses of 77 Gy and 94.1 Gy at α/β ratios of 3 and 1.5, respectively (in terms of 2 Gy per fraction). Therefore, our results align with previous evidence suggesting that higher bladder doses are key contributors to urinary toxicity, particularly in patient populations including high risk (low-, intermediate-, and high-risk population).

In addition to bladder dose constraints, the number of motion corrections was significantly associated with acute urinary toxicity (Table 1). To our knowledge, this study is the first to identify a relationship between motion correction and urinary toxicity. Motion correction appears to offer several benefits in reducing bladder dose.

First, motion correction involves recontouring the displaced target and reoptimizing the treatment plan. Although this process extends the overall treatment time, patient motion generally decreases as the session progresses, resulting in more stable positioning by the time irradiation begins. Consequently, during irradiation, patients who underwent motion correction exhibited smaller prostate displacements (Fig. 3a). Although not directly focused on the prostate, Honingh et al. reported that target motion was reduced in the latter half of the ART workflow compared with the initial half [24], suggesting that patients become more relaxed as treatment progresses. This observation, together with our finding that smaller prostate movements during irradiation were associated with reduced increases in bladder V37Gy (Fig. 3b), reinforces the notion that applying motion correction may contributes to minimizing bladder dose.

The interval between MRI acquisition and contouring (T1 in Supplementary Figure S3) was approximately 30 s longer when motion correction was applied than when it was not. Without motion correction in the ATS workflow, fusion and contouring can proceed immediately after pre-MRI acquisition. In contrast, when motion correction triggers the ATS workflow loop, target displacement should be verified on the PV-MRI after its acquisition. Then, the secondary re-fusion and recontouring are performed, resulting in further delay of approximately 30 s due to the position verification on the PV-MRI. However, secondary ATS-based recontouring (T2 in Supplementary Figure S3) took approximately 4 min and 30 s less time than the initial ATS-based contouring without motion correction. Thus, the overall interval between MRI acquisition and irradiation (T1–T3 in Supplementary Figure S3) was shorter when motion correction was applied (Fig. 4a). A shorter this interval was associated with a reduction in bladder V37Gy (cc) (Fig. 4b), underscoring the importance of minimizing this duration. Several studies have explored strategies to further reduce contouring time in ART. The ATS-light approach shortens contouring time by using propagated structures through rigid image registration (RIR) combined with ATS [25]. Because secondary ATS-based recontouring utilizes the most recent imaging as a reference for contour propagation, target propagation with RIR enables easy alignment. However, for the bladder, DIR is recommended to accurately capture its enlarged shape. Most OAR constraints can be satisfied using DIR-generated contours without manual modification [26]. When constraints are not met, typically at higher-dose levels [26], contouring time can be reduced by editing OARs only near the PTV. This workflow optimization can further decrease the MRI-to-irradiation interval.

Third, secondary ATS-based re-optimization to accommodate the enlarged bladder significantly reduced bladder V30–V37Gy (Table 3). Furthermore, replanning with ATS resulted in significantly lower bladder V30–V37Gy compared to replanning with ATP. In several institutions, secondary replanning triggered by motion correction is performed using the ATP process, which involves position correction and no plan re-optimization [8, 14, 27–30]. However, ATP cannot adjust the treatment plan to account for increased bladder volume during ART. Conversely, ATS allows plan modification to accommodate anatomical changes, reducing bladder V30–V37Gy even when the prostate remains stable (Table 3). Based on our findings, re-optimizing with ATS may minimize bladder dose in patients with substantial bladder filling or in cases where the bladder extends into the PTV. However, this analysis used a subset of 10 patients from the overall cohort of 114 patients. These patients were selected because they exhibited rapid bladder filling during ART workflow. This scenario is clinically relevant because bladder volume expansion may increase the risk of exceeding bladder dose constraints. Therefore, these cases were selected to evaluate the potential benefit of secondary ATS-based re-optimization. However, this selection may introduce selection bias, and the small sample size limits the generalizability of the findings. Thus, the results of this comparison should be interpreted with caution.

The Unity MR-Linac system includes a feature known as Comprehensive Motion Management (CMM) [31], which is not available at our institution. One of the key functions of CMM is baseline shift, which dynamically adjusts the multileaf collimator positions to match the displaced target during irradiation [32]. Because bladder dose increases as the target moves during irradiation (Fig. 3b), frequent positional adjustments using baseline shifts in CMM may be important to prevent acute urinary toxicity, even when the target remains within the PTV.

This study has several limitations. First, bladder wall dose constraints were not evaluated, although doing so may improve the accuracy of urinary toxicity prediction [14]. However, in the context of ART, using the entire bladder contour is more practical for clinical implementation, as it allows easier contour correction and dose accumulation. Second, the sample size was relatively small (*n* = 114); therefore, validation in a larger cohort is warranted to confirm the generalizability of these findings. Future analyses using larger datasets may enhance predictive accuracy through the integration of radiomics [33, 34] or deep learning–based radiomics features [35]. Third, only acute urinary toxicity could be assessed because the follow-up duration was insufficient to evaluate late toxicity. Nevertheless, several studies have reported that acute urinary toxicity is correlated with late toxicity [36–38], suggesting that patients who experience acute toxicity subsequently develop late effects. The fourth, the IPSS used in this study is a subjective patient-reported measure and may not directly correspond to physician-assessed acute toxicity based on the Radiation Therapy Oncology Group criteria. Finally, the calculation grid was set to 2.5 mm for high-risk patients and 3 mm for low- and intermediate-risk patients, resulting in patient cohorts with different calculation grid sizes. This difference may have introduced interpolation errors in the DIR and dose accumulation.

## Conclusion

In this study, cumulative bladder doses derived from pre-, PV-, and post-MRI were calculated to determine which timing was most closely associated with acute urinary toxicity. The findings indicated that cumulative bladder doses from PV- and post-MRI were more strongly correlated with acute urinary toxicity than those from pre-MRI. Specifically, bladder V37Gy (%) and V37Gy (cc) demonstrated associations with acute urinary toxicity in PV-MRI (AUC = 0.70 and 0.66, respectively) and post-MRI (AUC = 0.70 and 0.69, respectively). In addition, a higher number of motion corrections was statistically associated with reduced acute urinary toxicity (*p* = 0.04). Motion correction provided several advantages. First, secondary ATS-based recontouring and re-optimization associated with motion correction prolonged treatment time; however, prostate movement became more stable, resulting in smaller prostate displacements during irradiation. Second, secondary ATS-based recontouring required less time, shortening the interval between MRI acquisition and irradiation. Third, it enabled plan re-optimization based on the enlarged bladder anatomy. However, it needs to be careful that the correlation between motion correction and post-MRI bladder V37Gy (%) was weak (*r* = − 0.23), despite being statistically significant.

## Supplementary Information

Below is the link to the electronic supplementary material.

Supplementary Material 1

## Data Availability

Due to the restrictions of the institutional ethics board approval and patients’ consent, the raw imaging and clinical data cannot be publicly available. However, de-identified derived data supporting the findings of this study may be shared upon reasonable request, subject to approval by the institutional ethics board.
