# Supplementary material for: Optimal timing of the cumulative dose and factors related to acute urinary toxicity in prostate cancer treated using magnetic resonance linear accelerator
Source: Radiol Phys Technol. 2026 Apr 27;19(2):792–805. doi: 10.1007/s12194-026-01056-8 (PMC13253595; doi:10.1007/s12194-026-01056-8)
Supplement: Supplementary file 1 — Supplementary Material 1 [file 12194_2026_1056_MOESM1_ESM.pdf]

SUPPLEMENTARY INFORMATION

Optimal timing of the cumulative dose and factors related to acute urinary toxicity in prostate cancer treated using magnetic resonance linear accelerator

Shohei Tanaka, Ph. D.[1], Noriyoshi Takahashi, M.D., Ph. D.[1], Noriyuki Kadoya, Ph. D.[1], Wingyi Lee, M.S.[1], Taichi Hoshino, M.S.[1], Yoshiyuki Katsuta, Ph. D.[1], Kazuhiro Arai, Ph. D.[1], Yushan Xiao, M.S.[1], Hisamichi Takagi, Ph. D. [2], Yu Suzuki, M.D., Ph. D.[1], Shinsaku Okuda, M.D.[1], Keiichi Jingu, M.D., Ph. D.[1]

[1] Department of Radiation Oncology, Tohoku University Graduate School of Medicine, Sendai, Japan

[2] Department of Radiological Technology, School of Health Sciences, Faculty of Medicine, Tohoku University, Sendai, Japan

Corresponding author: Shohei Tanaka, Ph.D.

Department of Radiation Oncology, Tohoku University Graduate School of Medicine

1-1 Seiryo-machi, Aoba-ku, Sendai, 980-8574, Japan

Tel: +81-22-717-7312 Fax: +81-22-717-7316

E-mail: shohei.tanaka.a6@tohoku.ac.jp

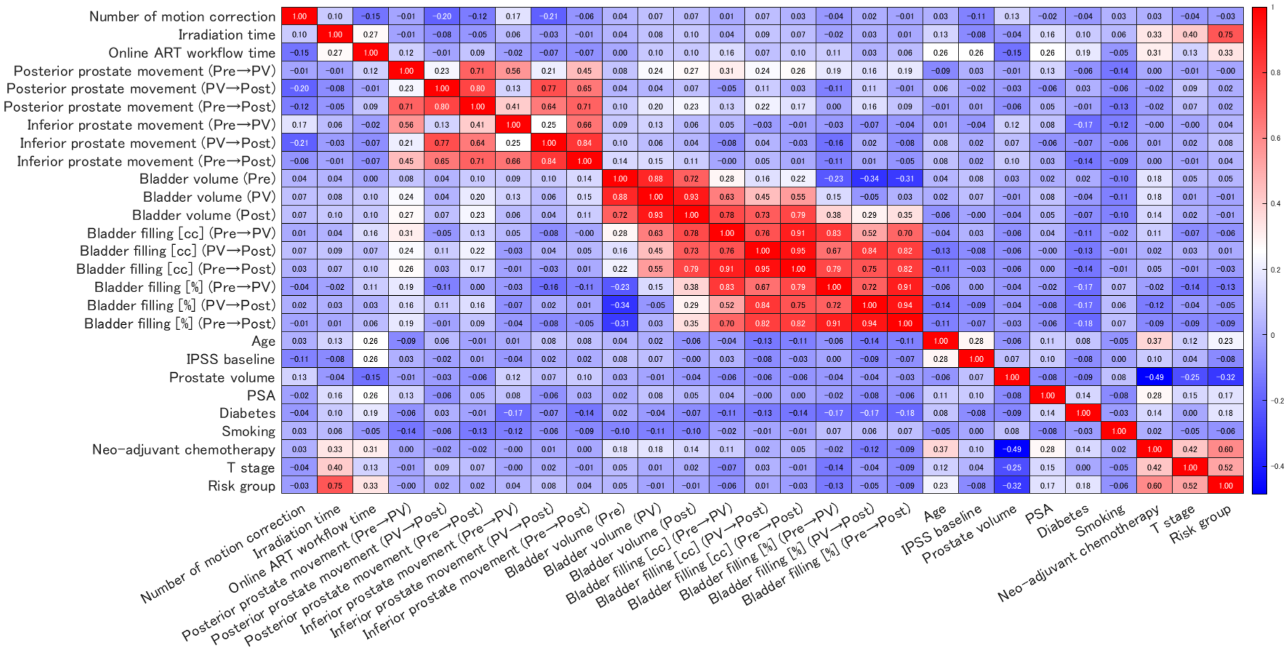

Supplementary Figure S1. All correlations between factors in the univariate analysis examining associations with acute urinary tract toxicity

Dark red indicates a strong positive correlation, dark blue indicates a strong negative correlation. “Number of motion correction” and “Bladder filling [%] (Pre–position verification (PV))”, which were associated with acute urinary tract toxicity in the univariate analysis, showed no correlation (r = −0.04).

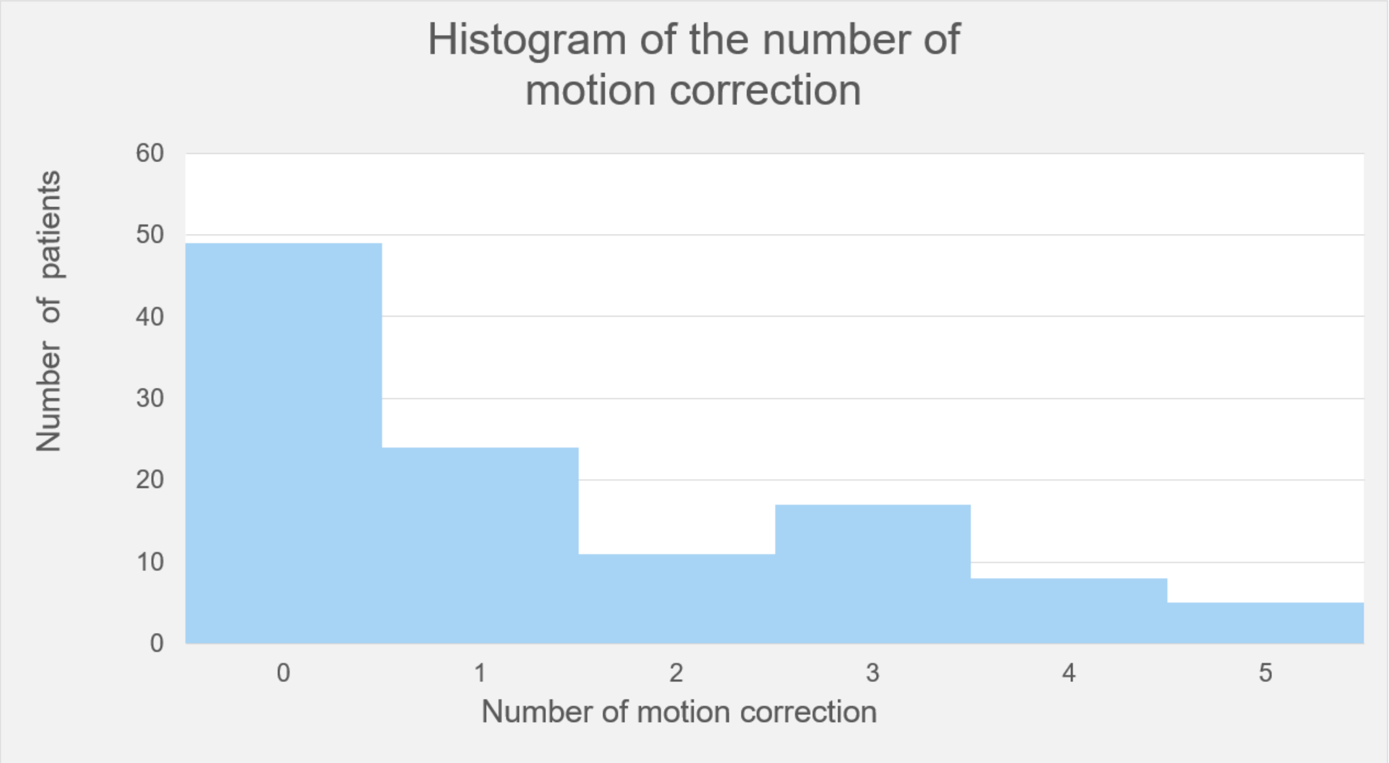

Supplementary Figure S2. A histogram of the number of motion corrections for all patients

The horizontal axis represents the number of motion corrections applied, and the vertical axis represents the number of patients. All patients (n = 114) received five-fraction treatment; therefore, the number of motion corrections ranged from 0 to 5. The largest group consisted of patients in whom motion correction was not applied in any fraction (0 times), comprising 49 patients. In contrast, only five patients received motion correction in all five fractions. The mean number of motion corrections per patient was 1.35, and the median was 1.

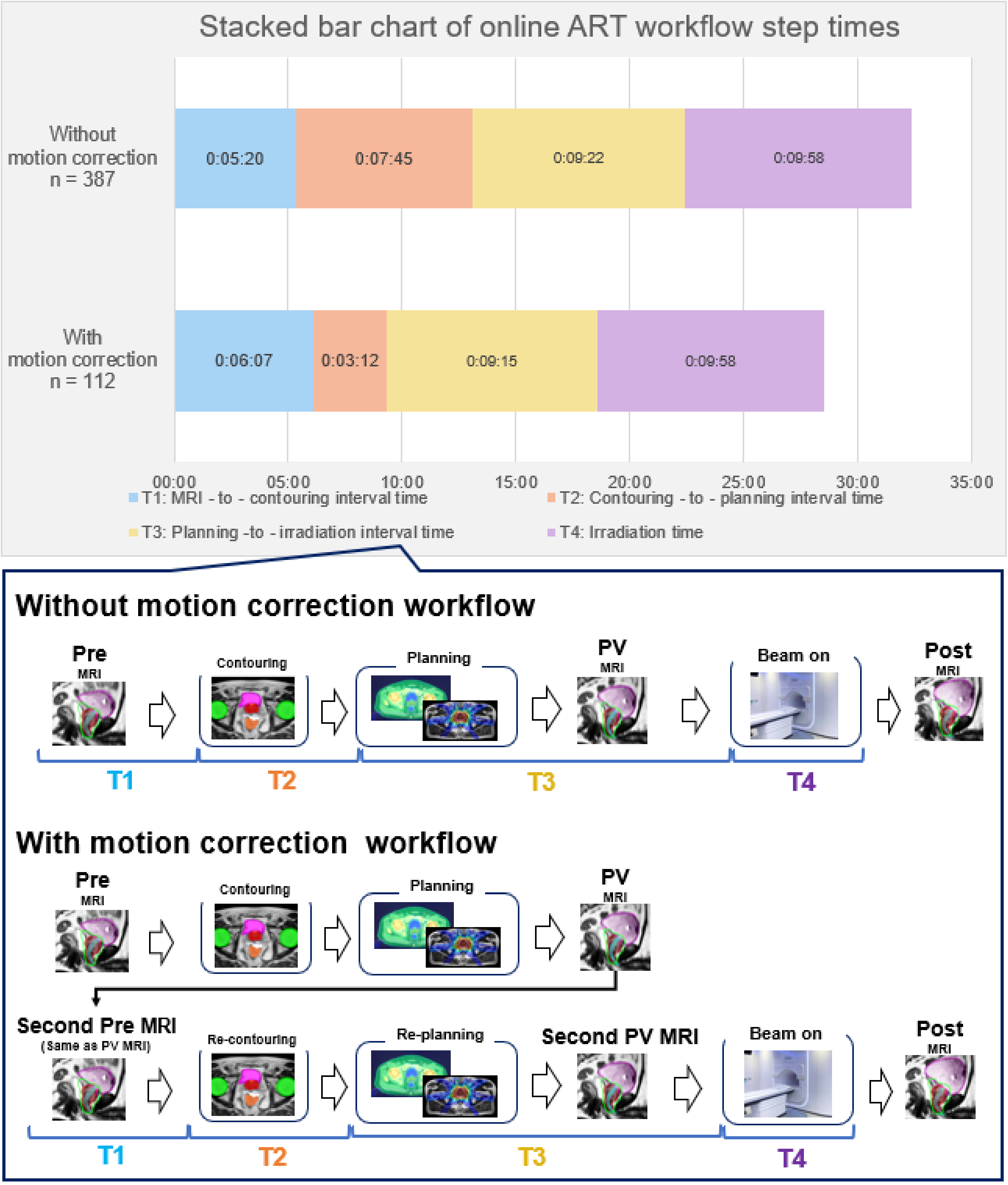

Supplementary Figure S3. Stacked bar charts of online adaptive radiation therapy workflow step times

Stacked bar charts show the time from magnetic resonance imaging (MRI) acquisition to contouring (blue, T1), contouring to planning (orange, T2), and planning to irradiation (yellow, T3) and the irradiation time itself (purple, T4) for fractions with and without motion correction. Only fractions treated using the adapt-to-shape workflow were included in the comparison. For fractions without motion correction, the online ART workflow time is measured from the first pre-MRI acquisition, while for fractions with motion

correction, it is measured from the second pre-MRI acquisition. When motion correction was applied, contouring time (orange, T2) was reduced, resulting in a shorter overall time from MRI acquisition to irradiation.

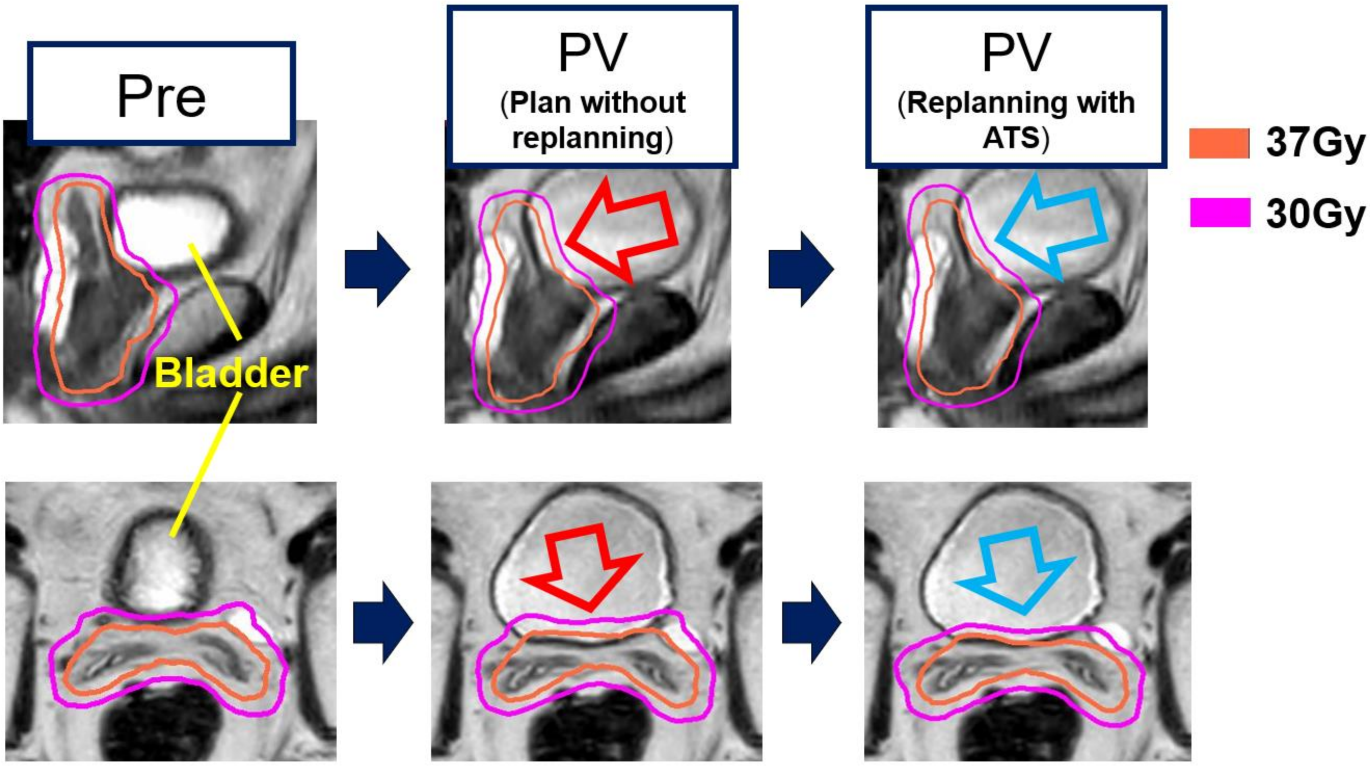

Supplementary Figure S4. Sagittal and axial images of a typical patient during pre-magnetic resonance imaging (MRI) and position verification (PV)-MRI with an enlarged bladder

The 30Gy and 37Gy (magenta and orange lines, respectively) were optimized to match the bladder in pre-MRI; however, the radiation dose to the bladder increased (red arrow) with the bladder volume in PV-MRI (plan without replanning). The irradiation dose to the bladder was reduced (blue arrow) through re-optimization (replanning with ATS).

Supplementary Table S1. Patient characteristics used in this study

Characteristic		n = 114
Age (years: mean±SD)		70.4 ± 7.1
Risk group classification	Low	35 (31%)
	intermediate	32 (28%)
	High	47 (41%)
T stage	1c	57 (50%)
	2a	29 (25%)
	2b	6 (5%)
	2c	7 (6%)
	3a	11 (10%)
	3b	4 (4%)
PSA (mean±SD)		11.2 ± 9.7
NeoAdjuvant chemotherapy	Yes	73 (64%)
	No	41 (36%)
Prescription	36.25Gy / 5fraction	67 (59%)
	40Gy / 5fraction	47 (41%)
IPSS baseline (mean±SD)		8.3 ± 5.8
Increase in IPSS (within 3 months)	<10	71 (62%)
	≥10	43 (38%)
SpaceOAR	Yes	114 (100%)
	No	0 (0%)
Bladder volume (cc: mean±SD)	pre-MRI	117 ± 56
	PV-MRI	150 ± 70
	post-MRI	202 ± 96

SD: standard deviation, IPSS: international prostate symptom score, PSA: prostate specific antigen, SpaceOAR: hydrogel spacer for organ at risk

Supplementary Table S2. Dose constraints for low- and moderate-risk groups with prostate cancer

Structure name		Optimal	Tolerable
PTV–rectum	D98%	>34.448 Gy	>32.625 Gy
	D95%	36.25 Gy	
	Dmax	<39.15 Gy	
PTV and rectum overlap	D98%	>34 Gy	>32.625 Gy
Rectum	V36.0 Gy	<1 cc	
	V32.6 Gy	<15%	
	V29 Gy	<20%	
	V25.3 Gy	<30%	
	V21.7 Gy	<40%	
	V18.1 Gy	<50%	
Bladder	V18.1 Gy	<40%	
	V37.0 Gy	<5 cc	<10 cc
Urethra PRV	V38.0 Gy	<0.1 cc	
Femur head	V14.5 Gy	<5%	
	Dmax	<25.375 Gy	

DXX%: dose administered to XX% of volume, Dmax: maximum dose of the volume, VXXGy: cc or percentage of volume receiving a dose≥ XXGy, PTV: planning target volume, PRV: planning organ at risk volume

Supplementary Table S3. Dose constraints for the high-risk group with prostate cancer

Structure name		Optimal	Tolerable
CTV–urethraPRV	D95%	>40 Gy	>39.2 Gy
PTV–rectum–urethraPRV	D98%	>34.448 Gy	>32.625 Gy
	D95%	36.25 Gy	
	Dmax	<43.2 Gy	
PTV and rectum overlap	D98%	>34 Gy	>32.625 Gy
Rectum	V36.0 Gy	<1 cc	
	V32.6 Gy	<15%	
	V29 Gy	<20%	
	V25.3 Gy	<30%	
	V21.7 Gy	<40%	
	V18.1 Gy	<50%	
Bladder	V18.1 Gy	<40%	
	V37.0 Gy	<5 cc	<10 cc
Urethra PRV	Dmax	<40 Gy	
Femur head	V14.5 Gy	<5%	
	Dmax	<25.375 Gy	

CTV: clinical target volume, PRV: planning organ at risk volume, DXX%: dose administered to XX% of volume, Dmax: maximum dose of the volume, VXXGy: cc or percentage of volume receiving a dose≥ XXGy, PTV: planning target volume

Supplementary Table S4. Factors causing MRgRT-specific anatomical changes

Factor	Description	Calculation method
Prostate movement	Inferior and posterior translations of the center of the mass of the prostate from pre- to PV-MRI, PV-MRI to post-MRI, and pre- to post-MRI.	Mean was calculated across five ART sessions per patient.
Bladder volume	Bladder volumes at pre-, PV-, and post-MRI.	Mean was calculated across five ART sessions per patient.
Bladder filling	Increase in bladder volume from pre- to PV-MRI, PV-MRI to post-MRI, and pre- to post-MRI.	Mean was calculated across five ART sessions per patient.
Irradiation time	Time required for irradiation.	Mean was calculated across five ART sessions per patient.
Online ART workflow time	Time from pre-MRI imaging to irradiation. In the standard ART workflow without motion correction, online ART workflow time was defined as the interval from the start of the first pre-MRI acquisition to the start of irradiation. In the online ART workflow loop triggered by motion correction, online ART workflow time was the interval from the start of the second pre-MRI acquisition (first PV-MRI) to the start of irradiation.	Mean was calculated across five ART sessions per patient.
Number of motion corrections	The number of processes for modifying the plan with additional ATS procedures for the moved target in PV-MRI.	Calculated among the five ART workflows (range, 0–5) because all patients with prostate cancer received five-fraction irradiation

MRgRT: magnetic resonance-guided radiation therapy, PV: position verification, ART: adaptive radiation therapy, MRI: magnetic resonance imaging, ATS: adapt to shape

Supplementary Table S5. Correlation between factors associated with acute urinary toxicity in univariate analysis and V37Gy

	V37Gy [cc] of post-MRI		V37Gy [%] of post-MRI	
	Spearman correlation coefficient	p value	Spearman correlation coefficient	p value
Number of motion corrections	−0.193	**0.039**	−0.226	**0.015**
Inferior prostate movement [mm] (PV→ Post)	0.456	**<0.001**	0.389	**<0.001**
Bladder filling [%] (Pre→PV)	−0.036	0.706	−0.196	**0.037**
Age	0.206	**0.028**	0.209	**0.026**
T stage	0.379	**<0.001**	0.363	**<0.001**

V37Gy: cc or percentage of volume receiving a dose ≥ 37Gy
